# Overcoming challenges to removing inappropriate penicillin allergy labels: A quality improvement report

**DOI:** 10.1017/ash.2021.171

**Published:** 2021-07-12

**Authors:** Risa N. Fuller, Mary Grace Baker, Mauli B. Desai, Patricia L. Saunders-Hao, Shradha Agarwal, Gopi Patel, Saloni Agrawal, Sarah H. Schaefer

**Affiliations:** 1 Division of Infectious Diseases, Department of Medicine, Icahn School of Medicine at Mount Sinai, New York, New York; 2 Division of Allergy/Immunology, Department of Medicine, Icahn School of Medicine at Mount Sinai, New York, New York; 3 Department of Pharmacy, North Shore University Hospital, Manhasset, New York; 4 Icahn School of Medicine at Mount Sinai, New York, New York

**Keywords:** Penicillin allergy, Antimicrobial stewardship, Quality Improvement

## Abstract

Over 3 months, we provided monthly education to internal medicine residents and distributed resources regarding penicillin-allergy history taking. Allergy information in the electronic record was updated more often during the intervention compared to the period before the intervention (16.1% vs 10.9%; *P* = .02). Education and interdepartmental collaboration have the potential to affect provider behavior.

Penicillin allergy is reported in 10% of the US population and in up to 15% of hospitalized patients; however, up to 95% of patients who report a penicillin allergy are found to be tolerant after testing.^1–3^ Patients who report a penicillin allergy are more likely to be treated with antibiotics that are broader in spectrum, more toxic, more expensive, and often less effective than the recommended first-line agents.^2^ As a result, these patients have increased rates of *Clostridioides difficile* infection, adverse drug reactions, and colonization with drug-resistant bacteria.^1,4^ Despite the well-demonstrated impact that a penicillin allergy label has on a patient’s care, providers often acknowledge that they have a limited understanding of the management of these patients.^5^

Herein, we describe a multidisciplinary quality improvement initiative implemented to improve provider understanding of penicillin allergy and to streamline inpatient allergy consultation. We aimed to facilitate the removal of inappropriate penicillin allergy labels, enabling proper antibiotic selection.

## Methods

**Figure 1.**
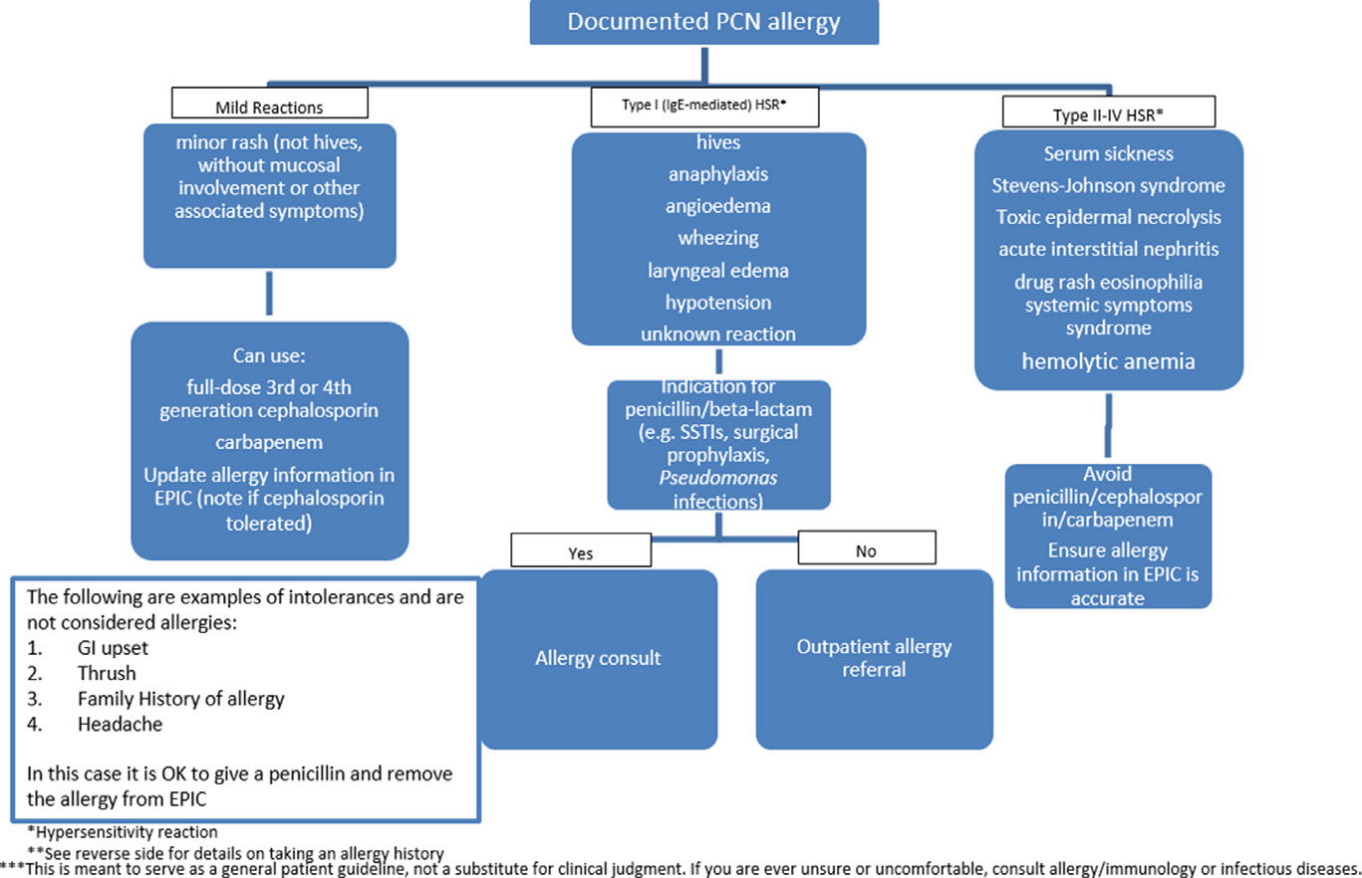
Allergy Algorithm

**Figure 2.**
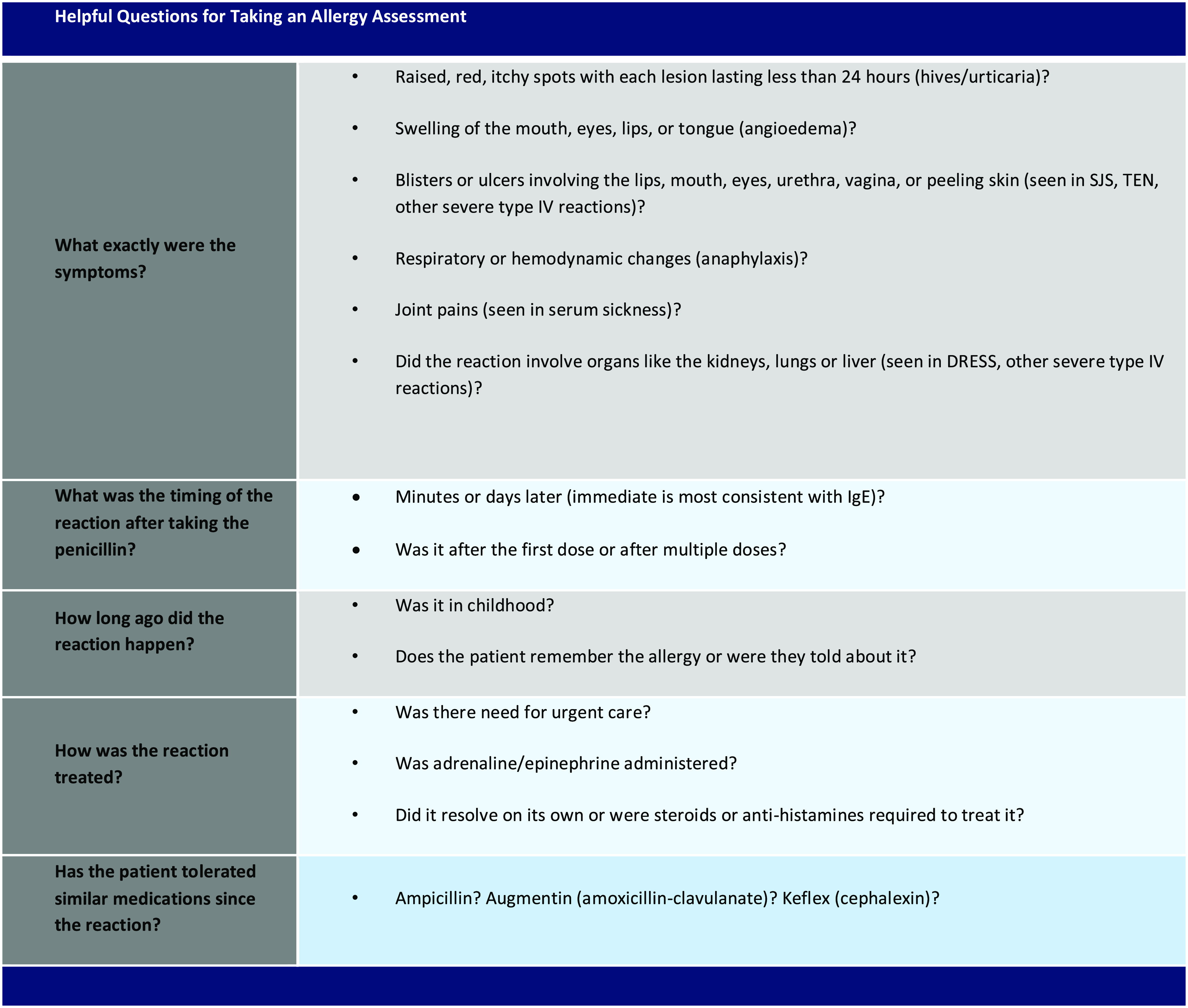
Allergy History-Taking Guide

Prior to our intervention, we organized a multidisciplinary team comprised of infectious diseases and allergy and immunology physicians and infectious disease clinical pharmacists. Based on our experience as front-line providers involved in antibiotic prescribing at our institution, we identified a gap in knowledge regarding penicillin allergies among the internal medicine residents. Through “Plan–Do–Study–Act” cycles, our team developed a guide for allergy history taking and an algorithm to direct next steps (Figs. 1 and 2). To streamline the process of inpatient allergy testing, the teams generated an order set in the electronic medical record (EMR; Epic, Verona, WI) inclusive of skin testing reagents, supplies, and anaphylaxis kits.

The initiative was implemented on the inpatient internal medicine service over the course of 3 months from November 1, 2018, through January 31, 2019. During this time, we attended the residents’ noon conference to provide a brief tutorial focused on the current literature, appropriate history-taking practices, and implementation of the algorithm. The resources were distributed on a reference card and were made available electronically through the residents’ smartphone application. The primary outcome measure was the frequency with which allergy information was updated in the EMR. This measure was felt to be the most direct indicator of the success of our education and the use of the algorithm. Secondary outcomes, analyzed on retrospective chart review, were type of antibiotic ordered and length of stay. We focused strictly on the penicillin allergy label and did not include other β-lactams because “penicillin allergy” is often used as an umbrella term encompassing several β-lactams. We used a 2-sample *z* test to calculate statistical significance. This initiative was deemed a quality improvement project by the Quality Improvement Committee in the Department of Internal Medicine at the Icahn School of Medicine at Mount Sinai. Outcomes during the 3-month intervention period were compared to the same 3-month period the year before (ie, preintervention period).

## Results

During the 3-month intervention period, there were 149 patient encounters on the internal medicine service for patients with a documented penicillin allergy. The median age of the preintervention cohort was 70 years, and the median age of the intervention cohort was 67 years (*P* = .26). Both cohorts were predominantly female: 69.1% of the preintervention cohort and 62.4% of the intervention cohort (*P* = .09). In the allergy section of our EMR, providers were able to characterize the reaction history by choosing from a list of signs and/or symptoms or by providing free text.

Prior to and during the intervention, the most common classifications selected included rash (27.3% preintervention vs 20.8% intervention), hives (7.9% preintervention vs 14.8% intervention), angioedema (1.8% preintervention vs 1.3% intervention), anaphylaxis (5.5% preintervention vs 9.4% intervention), or “unknown” (21.2% preintervention vs 15.4% intervention). Following the intervention, the frequency with which a reaction was classified as “unknown” decreased significantly (21.2% preintervention vs 15.4% intervention; *P* = .04). Allergy information in the EMR was updated 10.9% of the time in the preintervention cohort and 16.1% of the time in the intervention cohort (*P* = .02).

Although we did not directly track who updated the allergy information, we presume that the updates were performed by residents because they are the providers primarily responsible for admitting patients, updating the medical history including allergies, and documenting in the EMR. Allergy consultation and testing were not performed on any of these patients. During the intervention period, 66% of these patients received inpatient antibiotics. Of these, 64.3% received a β-lactam during their hospital stay despite having been admitted with a penicillin allergy label. There was no significant increase in β-lactam use compared to the preintervention period (72.7% vs 64.3%; *P* = 1.00). We detected a statistically significant decrease in aztreonam use in the intervention group compared to the preintervention group (5.1% vs 11.8%; *P* = .02). There was no significant change in the frequency of use of any other antibiotic. The median length of stay was 6 days in the preintervention cohort and 4 days in the intervention cohort (*P* < .001).

## Discussion

Penicillin allergies are common in hospitalized patients. These labels are often incorrect, and perpetuating this allergy label is not benign.^6^ Through provider education and distribution of an algorithm, we were able to change provider behavior, demonstrated by the fact that providers were significantly more likely to update a patient’s allergy history in the EMR during the intervention period and that significantly fewer reaction histories were classified as “unknown.” Algorithms have previously been shown to be useful in reinforcing safe practices regarding the use of β-lactam antibiotics in penicillin-allergic patients.^2,7^

Skin testing was not utilized during our study period. Our population likely represents the “low hanging fruit”of penicillin allergy labels, for whom history-taking alone can successfully exclude intolerances and other nonallergic reactions. Many institutions do not have access to an inpatient allergy and immunology service or the resources to perform skin testing in the inpatient setting; thus, the ability to successfully address a subset of these mislabeled patients is critical to the delivery of safer patient care. Although there was no significant change in β-lactam usage between the 2 cohorts, the intervention period was associated with a significant decrease in aztreonam use, a monobactam antibiotic commonly used as an alternative in penicillin-allergic patients. A decrease in aztreonam usage is associated with significant cost savings.^8^ Removal of inappropriate penicillin allergy labels also facilitates an easier transition to oral antibiotics, a key component of antibiotic stewardship programs that can aid in more timely hospital discharge.^6,9^ Although many factors contribute to a patient’s length of stay, we did note a decreased median length of stay during the intervention period.

This study has several limitations. We were not able to directly link the demonstrated change in provider behavior with patient outcomes due to the brief nature of study. The modification in resident behavior during the study period may have been due to the Hawthorne effect, so future research should include long-term data to determine whether the impact of education is sustainable. However, this initiative can be expanded to other populations, such as oncology patients, who have high antibiotic utilization and historically poor antimicrobial stewardship.^9^

In conclusion, we have demonstrated that education and interdepartmental collaboration have the potential to affect provider behavior when it comes to taking an allergy history and appropriate antibiotic selection for patients with a penicillin allergy label. The impact of this initiative may be even greater when conducted over a longer period, accompanied by interval re-education, and this intervention may be expanded to other patient populations.
